# Self-Screening for Cervical Cancer Offered through a Digital Platform in a Region of British Columbia with Lower Screening Rates

**DOI:** 10.3390/curroncol31090399

**Published:** 2024-09-13

**Authors:** Laurie W. Smith, Amy Booth, C. Sarai Racey, Brenda Smith, Ashwini Prabhakaran, Smritee Dabee, Quan Hong, Nazia Niazi, Gina S. Ogilvie

**Affiliations:** 1 Women’s Health Research Institute, Vancouver, BC V6H 3N1, Canada; 2 BC Cancer, Vancouver, BC V5Z 4E6, Canada; 3 School of Population and Public Health, University of British Columbia, Vancouver, BC V6T 1Z3, Canada; 4 Island Pre-Health Science Program, North Island College, Courtenay, BC V9N 8N6, Canada; 5 Surrey-North Delta Division of Family Practice, Surrey, BC V3S 5A5, Canada; 6 BC Centre for Disease Control, Vancouver, BC V5Z 4R4, Canada

**Keywords:** cervix screening, human papillomavirus, HPV testing, self-screening, digital health

## Abstract

Cervical cancer is highly preventable through vaccination, early detection, and treatment, yet is the fourth most common cancer globally. HPV testing is superior to cytology for the detection of cervical pre-cancer, and jurisdictions around the world are implementing HPV primary screening, which offers the opportunity for self-screening, an important self-care intervention. Digital health solutions are also increasingly important components of self-care. In this study, we assessed the acceptability and completion of self-screening for cervical cancer offered through a digital platform within a low screening uptake region of British Columbia. The primary objective of this study was to evaluate the acceptability of self-screening for cervical cancer offered through a digital platform as measured by return rates of self-screening kits. Patients due or overdue for cervix screening were invited to participate. Eligible participants registered online to receive a self-screening kit, which included a device for vaginal self-screening, instructions, and a return envelope, sent to their home. After self-screening using the vaginal device, HPV testing was conducted. HPV-negative participants were returned to routine screening, and HPV-positive participants were recommended for cytology or colposcopy. Attendance rates at follow-up were evaluated. Participants were invited to complete an acceptability survey. From April 2019 to December 2023, 283 participants were sent kits, with 207 kits returned for a completion rate of 73%. Of valid samples (n = 202), 15 were HPV positive, and 93% attended follow-up care. Most respondents found the CervixCheck website easy to use, informative, and secure and were satisfied with receiving their results online. CervixCheck had a high completion rate among participants who were sent a self-screening kit. High compliance with recommended follow-up and high acceptability of self-screening for cervical cancer was observed. Most participants indicated they would self-screen again in the future. Innovative approaches to cervical screening, including self-screening and the use of digital health interventions, are ways to enhance equity and improve uptake of cervical screening.

## 1. Introduction

Cervical cancer is the fourth most common cancer in women worldwide, despite being almost entirely preventable through HPV vaccination and early detection and treatment with screening [1]. For decades, cervical cancer screening with the Pap test has been considered a public health success, decreasing cervical cancer morbidity and mortality significantly [2]. However, in Canada, the incidence rate of cervical cancer has been increasing by 3.7% per year since 2015, and it is now the fastest-increasing cancer for Canadian females, with similar increases seen in countries worldwide [3]. In 2020, the World Health Organization released a global strategy for the elimination of cervical cancer as a public health concern by 2030, with a targeted global incidence rate of 4.0/100,000 females [4]. In 2020, the incidence rate of cervical cancer in Canada was an estimated 7.1/100,000 females [5]. Following the announcement of the WHO strategy, the Canadian Partnership Against Cancer (CPAC) developed an Action Plan for the Elimination of Cervical Cancer in Canada by 2040 [6]. Both the WHO and CPAC elimination strategies are anchored in three pillars: (1) improve HPV vaccination rates; (2) implement HPV primary screening; and (3) improve follow-up of screening results [4,6]. 

It is now well established that HPV testing for cervical screening is superior to Pap test cytology for earlier detection of cervical pre-cancer [7,8,9]. In recent years, regions around the world have begun the implementation of HPV primary screening, including Australia, the Netherlands, Denmark, Sweden, and the United Kingdom. Since HPV testing does not require a sample of cervical cells, HPV testing presents the opportunity for samples to be collected by providers (cervically or vaginally) or by individuals themselves (vaginally) [10]. In Canada, two provinces have transitioned to HPV primary screening: Prince Edward Island and British Columbia (B.C.). B.C. commenced the transition in January 2024, with widescale offering of self-screening for anyone between the ages of 25 and 69 due and eligible for average-risk cervix screening. The BC Cervix Screening Program was the first organized screening program in Canada to transition from Pap test cytology to HPV primary screening, with self-screening available for anyone who is eligible for screening and would prefer to self-screen. 

Around the world, self-care interventions are increasingly recognized as innovative strategies that make healthcare more accessible and equitable [11]. Self-screening for cervical cancer, as an alternative to provider-collected sampling, is an empowering self-care intervention that has the potential to increase access for those who may have historically faced challenges and barriers with provider-collected screening [12,13,14]. Globally, research has found that self-screening is acceptable and increases screening uptake, especially in never-screened or overdue-for-screening women and individuals with a cervix [15,16,17]. In addition, self-screening is a viable option during times when access to providers may be challenging (e.g., during global pandemics, in conflict zones, during acute climate crises, or when access to primary care providers is limited). 

The use of digital health applications to improve access to health services and care is increasing around the world and is an important aspect of self-care [11]. For example, internet-based services have been successfully implemented in the field of sexual health [18,19]. Like self-screening, internet-based services improve access for people facing barriers and challenges to accessing in-person care with a provider [19] and can increase autonomy. Furthermore, digital health applications are shown to be acceptable across a wide variety of demographics [20,21]. 

Modeled upon the success of a web-based approach for testing for sexually transmitted and blood-borne infections (STBBIs) in B.C., we developed an innovative approach to offer self-screening for cervical cancer through an online platform within a region in B.C. with a low uptake of provider collected cytology screening. The objectives of this study were to assess the acceptability and completion of self-screening for cervical cancer offered through a digital platform and to assess adherence to recommended follow-up for HPV-positive participants within a low-screening region of British Columbia.

## 2. Materials and Methods

### 2.1. Study Design

The primary objective of this observational study was to evaluate the acceptability of self-screening for cervical cancer offered through an online website, called CervixCheck, as measured by the return of completed self-screening kits. The novel, innovative digital health solution was modeled on a similar health service available in British Columbia: GetCheckedOnline (GCO) [19], a web-based platform offering testing for STBBIs. Using the GCO model, a website was specifically designed to offer at-home self-screening kits for cervical cancer, available to patients due or overdue for cervix screening. 

This study was designed in partnership with collaborating family physicians in a region of B.C. with the lowest provider-collected cytology screening participation rates in the province [22]. CervixCheck was offered through eight collaborating family medicine clinics in a region within the South Surrey Division of Family Practice in Southwest, B.C., which serves predominately South Asian patient populations. Through the CervixCheck website, individuals could register online to receive an at-home self-screening kit sent to them at an address of their choice (typically a home address). Ethics approval was obtained from the University of British Columbia Clinical Research Ethics Board (H18-00511) and online consent was obtained from all participants.

### 2.2. Participant Recruitment 

Between April 2019 and December 2023, collaborating physicians invited patients from their practices to participate in CervixCheck if they had not been screened for cervical cancer in three or more years, were 25–65 years of age, and had provincial healthcare coverage in B.C. Physicians provided patients with a brief introduction to self-screening and referred them to the CervixCheck website for further information. The CervixCheck website included background information on HPV, cervical cancer, the advantages of screening, the self-screening process, and the potential follow-up pathways, depending on HPV testing results. Given that CervixCheck was conducted in busy family practice clinics and the clinicians and office staff were resource strained, particularly, during the COVID-19 pandemic, the total number of patient encounters where patients were provided with CervixCheck information and the option to participate was not monitored.

### 2.3. Eligibility and Intervention

Participants received an access code to register for the CervixCheck website from the collaborating physicians or through advertisement posters. Registration for CervixCheck required a valid email address and provincial personal health number (PHN). As part of the account registration, participants reviewed an electronic consent form and were instructed to self-exclude if they were pregnant, were HIV positive, had a total hysterectomy, and/or were receiving immunosuppressive therapy. After consenting and creating an account, the participant’s B.C screening history was reviewed by authorized study staff via provincial screening records. This study staff confirmed study eligibility if the participant’s B.C. screening record indicated no recorded cervix screen in the last 3 years (the interval for average risk screening in B.C at the time of study recruitment), no history of invasive cervical cancer, and no history of cervical intraepithelial neoplasia grade two or higher (CIN2+) in the last 5 years.

Eligible participants received a cervix self-screening kit in the mail, which included a vaginal self-screening device, instructions for self-screening, and a prepaid envelope for returning the sample to the B.C. cervical cancer screening lab (CCSL). Two dry-collected vaginal self-screening devices were used for screening over the course of this study: the HerSwabTM (Eve Medical, Toronto, ON, Canada) and the COPAN 552C.80 FLOQSwab^®^ (Copan Italia s.p.a., Brescia, Italy). The HerSwabTM was used from April 2019 to December 2021 and, after this device was discontinued, the FLOQSwab was introduced. Any participants with outstanding HerSwabTM devices after December 2021 were sent a replacement FLOQSwab device to use. Previous studies have used both these devices and found high sensitivity and patient acceptability [23,24,25,26]. Participants were sent up to three email reminders to return their self-collected sample kit. The first email reminder was sent approximately 3–4 weeks after the kit was mailed to the participant, the second reminder was sent at approximately 6–8 weeks, and the final reminder was sent approximately 5–6 months after the initial mailing for the self-screening kit. 

### 2.4. Outcome Measures

The primary outcome of this study was to evaluate the completion rate of self-screening through the proportion of returned self-screening kits after registration and request on the CervixCheck website. Secondary outcome measures included attendance rates at follow-up among participants who tested positive for HPV after self-screening, time to self-screen completion after the kit was sent, and acceptability of self-screening and the online platform as measured through a follow-up questionnaire among those who completed self-screening. 

### 2.5. HPV Testing and Results Dissemination 

The self-collected vaginal devices were sent to the Cervical Cancer Screening Lab (CCSL) by the participant, where samples underwent HPV testing. At the CCSL, the dry-collected samples were eluted in PreservCyt^®^ medium (Hologic, Marlborough, MA, USA), and HPV testing with partial genotyping was performed using the Roche cobas^®^ 4800 (Roche Diagnostics Canada, Laval, QC, Canada). HPV genotypes 16 and 18 were identified and reported individually; other high-risk HPV genotypes were identified as a pooled result (31, 33, 35, 39, 45, 51, 52, 56, 58, 59, 66, 68). The HPV result was reported as invalid if human beta-globin was not detected, indicating insufficient cellularity required for testing. If a sample arrived at the lab more than 28 days after collection, the sample was rejected, and the participant was contacted with the option to re-collect. 

Self-screen HPV results were reported via an electronic medical record system to both the participant’s family physician and the study centre. Study staff were responsible for updating the participant’s account on the CervixCheck website to initiate an email notifying the participant that their result was available on the CervixCheck website. When a participant was HPV negative, they were informed of their result through their secure account on the CervixCheck website. When an HPV-positive result was reported (HPV 16, HPV 18, or other high-risk HPV positive), participants were instructed to contact their family physician for their results and recommended follow-up. When an HPV-invalid result was reported, participants were informed of a sample processing issue through their secure CervixCheck account and subsequently contacted by study staff with the option to re-collect.

**Figure 1:**
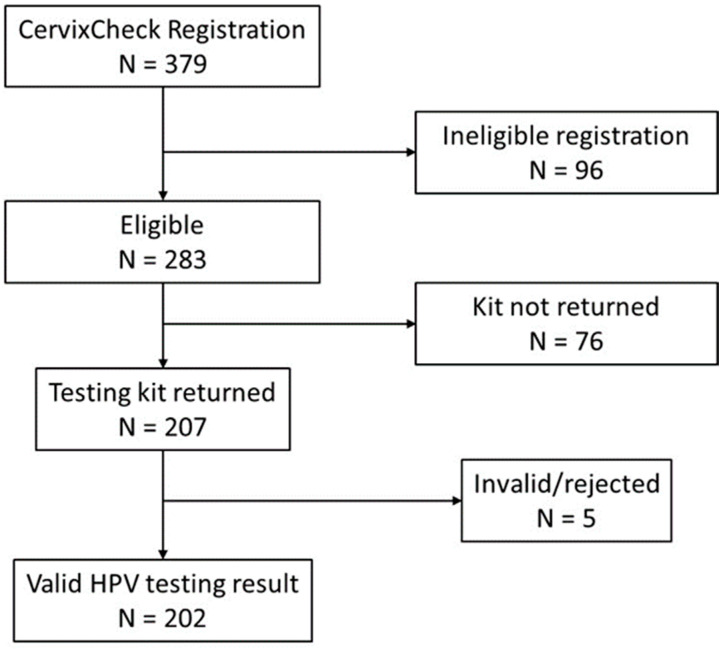
Participation in CervixCheck. Any participants who withdrew from this study are not included in this analysis.

The management of HPV results and recommendations for follow-up management were determined based on the standard of care in B.C. and advice from the Medical Director of the Cervical Screening Program (Figure 1). At the time of this study’s conduct, the standard of care in B.C. was cervical cytology at 3-year intervals for average-risk individuals with negative screening results. 

CervixCheck participants with a negative self-screen HPV result were recommended to return to regular cervix screening per the standard of care, as recommended by the BC Cancer Cervix Screening Program. Participants with positive HPV 16 and/or 18 results were referred directly to colposcopy, without additional triage. Those positive for other high-risk (OHR) HPV types were recommended to see a healthcare provider for cervical cytology collection to determine further management. Those OHR HPV positive with negative cytology results at month 0 were recommended to have follow-up cytology at 12 months and subsequently at 24 months if the 12-month cytology was also negative. Those with low-grade grade dysplasia or higher (ASCUS or greater) at their 0-month reflex cytology or at their 12-month or 24-month cytology were referred to colposcopy. Participants initially positive for OHR HPV required three consecutive negative cytology results to be returned to regular screening.

### 2.6. Post-Participation Survey

All eligible participants who registered for CervixCheck and consented to future research were invited to complete an online post-participation survey about their experiences with the online platform and with HPV self-screening for cervical cancer (Supplementary Materials). Simple demographic information (language, ethnicity, household income) was also collected. Invitations to complete the survey were sent after participants received notification of their HPV results or after three reminders to return kits were sent (i.e., post-participation surveys were sent to those who returned a completed self-screening sample and those who did not). If the participant did not open the individualized survey link after two weeks, one reminder email was sent. Questions in the survey included reasons for participating in CervixCheck, ease of using the website, concerns around data privacy and security, experience with self-screening, acceptability of receiving results online, and preference for future screening. The survey was designed, piloted, and administered using the online survey tool REDCap 12.0.30 [27]. The survey was piloted amongst approximately 20 females of screen-eligible age to obtain feedback on the length of the survey, comprehension of the questions, and clarity of the language.

## 3. Results

### 3.1. Self-Screening Completion

From April 2019 to December 2023, a total of 379 people registered on the CervixCheck website and self-determined eligibility for participation, of which 283 (74.6%) were due for cervix screening and mailed a self-screening kit (Figure 1). Of those sent a kit, 207 (73.1%) returned the kit to the provincial laboratory for HPV testing. The median time from the kit being sent to vaginal sample collection was 22 days (IQR 10–48). Most participants required no (42%) or only one (35%) reminder before returning their kits. No samples arrived at the lab greater than 28 days after collection.

Overall, there were no significant differences between individuals who did and did not return the self-screening kit (Table 1). The median age of those who returned the kit was 45 years (IQR 37–53), and the median age of those who did not return the kit was 44 years (IQR 38–51). Most participants who returned a kit (67.6%) were due for screening (3–4 years since last screen and not overdue), and the median time since last screen was 3.9 years (IQR 4.4–5.0). Of those returning a kit, 22.2% had gone 5 or more years since their last screening, and 10.1% had no history of screening. 

The majority of those who did not return a kit (69.7%) had a screen within the last 4 years. Of those who did not return kits after registering, 19.7% had gone 5 or more years since their last screen, and 10.5% had no history of screening. Of registered participants who did not return the kit, 56.6% subsequently received a cervix screen through the provincial program at the time of data analysis. Bivariable comparisons between those who returned and did not return kits show no differences in age at the time that the kit was sent (*p* = 0.55) or time since their last screen (*p* = 0.82).

For those returning kits, 202 (97.6%) yielded a valid result and 2.4% were invalid or rejected, and these participants did not subsequently return a valid kit. There were 15 (7.4%) participants with HPV-positive results (HPV 16, HPV 18, and/or OHR-HPV) (Table 2), of which 14/15 (93.3%) attended recommended follow-up (colposcopy or Pap).

### 3.2. Survey Results

There were 224/283 (79.1%) CervixCheck registrants (who returned or did not return kits) who consented to future research and were invited to complete feedback surveys, of which 51 (22.7%) responded (44 complete and 7 incomplete surveys) (Table 3). Only those who returned kits and fully completed the survey (n = 42) are included in the demographic analysis (Table 3). The majority of participants who completed the surveys were Asian (59.5%) and the majority were comfortable speaking English (83.3%). Almost half of the respondents (48.3%) had a household income over CAD 90,000 CAD per annum. 

Participants were asked to describe their experience using the CervixCheck website through a variety of questions (Table 4). When asked about the CervixCheck website, 93.5% indicated creating an account was easy to do, 84.1% indicated the website was easy to use, 88.4% indicated that the language was easy to understand, and 81.4% were satisfied with the content and features of the website. Furthermore, the majority of participants (83.7%) felt confident about the confidentiality and privacy of the website. Those who completed self-screening were asked about their experience receiving screening results online; 86.8% felt satisfied with the receipt of results online and 92.1% reported that they understood their online results.

Participants who returned kits were given the option to indicate their reasons for participating in this study, with the most common reasons being convenience (52.9%) and reduced discomfort compared to a Pap smear (43.1%). When asked more specifically about the experience of self-screening, 92.1% reported that the vaginal device was comfortable to use, that there was no discomfort, and that the instructions were easy to follow; 86.8% reported that self-screening was easy to perform and that they felt they were collecting the sample correctly. A large majority (92.1%) indicated they were likely to use self-screening again in the future, and 84.2% would prefer self-screening over provider-collected screening. 

Survey respondents were also provided with the opportunity to provide open-text comments regarding their experiences as a participant in the CervixCheck study. Among “Other” reasons for participating in CervixCheck, participants reported “Due to COVID-19” and “Suggested by doctors”. Participants also had the following to say about the CervixCheck website: “It was easy to use” and “More women will get screened when they can do so from the comfort of their own homes”. Finally, participants shared the following about the self-screening process: “I have shared my experience with other women, and they are excited of this possibility” and the “Process was easy and painless”.

## 4. Discussion

CervixCheck, a digital application for primary self-screening for cervical cancer, had a completion rate of 73% among participants who registered and were eligible to receive the self-screening kit. There was high compliance with recommended follow-up at 93% and high acceptability of self-screening, with 92.1% of users reporting they would self-screen again in the future.

This analysis presents findings from an observational study conducted in British Columbia, evaluating the uptake and acceptability of self-screening for cervical cancer offered through an online platform. Research has demonstrated that cervical screening is higher amongst those offered self-screening compared to those only offered provider-collected screening, regardless of the invitation method [14,28]; however, opt-in strategies, where individuals are invited to request a kit, are less effective than opt-out strategies, where individuals are automatically sent kits and do not need to formally request them [14]. In our study, using an opt-in method, 73% of those eligible who created an online account through CervixCheck to receive a self-screening kit returned their self-screening kit. Return rates of self-screening kits with an opt-in approach are variable. Other findings have reported similar return rates after kit requests of 65% to 80% [29,30,31]. The reported participation rate for cervix screening in this region of British Columbia is 63% [22]. With a completion rate of 73%, our findings indicate that self-screening may be a low-barrier alternative to improving participation rates in this region. 

We found that most participants required only one or two reminders before returning their kits and that the majority of them returned their kits to the lab less than 30 days after sample collection. This information can be useful for programs planning for the implementation of a mailed-out approach for self-screening kits and for understanding the variability of participant behaviour and the use of reminders within a program setting. In addition, understanding kit return rates and time to return kits will also inform laboratory planning, with some insight into resources required for sample processing with self-screening for cervical cancer. 

Those overdue or never screened for cervical cancer are at the highest risk for cervical pre-cancer and cancer [3,32,33]. Of the CervixCheck participants who registered for an online account and returned kits, 32% were 5 or more years overdue for screening or had no history of cervical screening. In B.C., 66% of those with invasive cancer are 5 or more years overdue or have no history of screening [22]. A digital health approach, like CervixCheck, providing a low-barrier option with a high-performance screening test to those at highest risk for developing cervical cancer, may be an effective way to improve screening uptake and accelerate the elimination of cervical cancer. 

Of those who registered and requested to receive a self-screening kit, 27% did not return their self-screening kits despite several attempts to reach them. However, 57% of those who requested but did not return a kit attended provider-collected screening by the time this analysis was completed. Although they did not return the kits, it is plausible that receiving a kit and engaging with the CervixCheck website about cervical cancer screening prompted them to attend provider-collected screening. 

For the prevention of cervical cancer through screening to be effective, it is important to increase participation in screening, and it is critical that those with non-normal or high-risk HPV test results attend recommended follow-up. Among CervixCheck participants who were HPV positive, there was high attendance at follow-up, with 93% of those recommended to receive follow-up attending the initial recommended follow-up appointment. The vast majority of HPV-positive participants required cytology with a provider as their recommended follow-up. One documented concern with offering self-screening for cervical cancer is the potential for loss to follow-up after a positive result [34]. The barriers that screening women and individuals with a cervix face with the traditional approach for cervical screening (i.e., the need to see a provider for a gynecological exam) are removed with a self-screening approach but are re-introduced when follow-up is required for HPV-positive self-screening results. In our study, compliance with recommended follow-up was high, indicating that those who screen positive may be more motivated to attend an in-clinic visit with a provider after receipt of a positive result [35].

In examining the acceptability of CervixCheck as a digital health application for self-screening, it is important to separate the participant’s acceptability of and user experience with the digital application and that of the process of sample collection for self-screening. As part of the feedback survey, participants provided feedback regarding their experience using the website. Of those who returned a kit and completed the survey, feedback regarding the website was positive. Over 84% indicated that the website was easy to use and that it provided enough information about participation. When asked about website confidentiality, 84% felt confident about the confidentiality and privacy of CervixCheck, and 87% of respondents felt satisfied with receiving their results online. Some of the best experiences with a self-care intervention offered through a digital health approach have occurred with sexually transmitted and blood-borne infection (STBBI) testing and treatment. Accessing testing and treatment for STBBIs through online services is increasingly available and has been shown to increase the uptake of testing and treatment in a variety of settings [18,19]. In settings where access to providers may be limited or challenging, online approaches to accessing healthcare are increasingly important. The success of internet-based approaches in STBBI care may also be applied to self-screening for cervical cancer. As more jurisdictions implement self-screening as part of a primary HPV screening program, offering internet-based kit requests and distribution may be an approach to improve coverage and enhance equity. 

Participants who returned their self-screening kits and completed the survey also reported high levels of acceptability to the self-screening sample collection. Over 86% of participants found the self-screening device easy to use and that they collected the sample correctly. Moreover, 92% of participants indicated they would recommend self-screening to friends and family. This is consistent with previous work on self-screening devices, which has overwhelmingly found that individuals find the collection of self-screening samples to be acceptable [15,16,17].

### Limitations

When interpreting the results of this observational study, there are limitations to be considered. Recruitment into this study was primarily conducted by collaborating physicians and clinic staff, and the number of patients approached to participate was not recorded. Therefore, we do not have a reliable estimate of how many patients declined participation in this study, including registration on the CervixCheck website. However, this is also a reality of conducting research in the community-based setting, particularly in busy, resource-strained family practice clinics. In addition, the significant majority of surveys were completed by those who returned the self-screening kit, and as such, we have minimal insight into why participants chose not to complete self-screening through CervixCheck after requesting a kit or their perspective on the acceptability of self-screening for cervical cancer. 

The survey and the website were only available in English, decreasing the accessibility of the service for non-English speakers and readers. A majority of CervixCheck participants were recruited from a single-family practice rather than equal distribution across the collaborating family practices. We suspect this is because of increased physician endorsement and engagement in the practice that recruited the most participants, which may have also impacted the likelihood of returning the kit and the overall acceptability of cervix screening. However, this practice is also one of the busiest in the region, conducting significantly more cervical screens than other practices, and many other providers in the region refer their patients to this clinic for cervical screening. Another limitation of this study is that it was conducted in collaboration with patients attached to primary care providers; as such, we cannot draw strong conclusions about the acceptability of CervixCheck as a digital health application for HPV self-screening among unattached patients.

There are also several strengths of this study. This study was conducted in a community setting in busy family practices, reflecting the real-world experience of cervical screening in the primary care setting. In addition, this study was conducted during the global COVID-19 pandemic and provided an opportunity for those due for screening to receive screening during a time when non-essential services were limited or not available at all. This study was also conducted in collaboration with the BC Cancer Cervix Screening program, one of the longest-standing organized screening programs in the world. Lessons learned from this study have informed the provincial roll-out of primary screening in B.C., where the screening program has included an electronic kit request option for the self-screening program.

## 5. Conclusions

HPV primary screening is a more effective way to achieve the elimination of cervical cancer than cytology. However, improved testing technology alone will not achieve elimination. Innovative approaches to offering HPV primary screening, including self-screening, can move us closer to the Canadian and global elimination goals. In addition, giving women and individuals with a cervix choice in how they access and receive cervical screening will enhance equity and increase uptake of cervical screening and attendance at follow-up.

## Figures and Tables

**Table 1:** Characteristics of CervixCheck participants receiving a self-screening kit in the mail.

	Total Eligible (N = 283)	Kit Returned (N = 207)	Kit Not Returned (N = 76)	*p*-Value *
Age when the kit was sent (median, IQR)	45 (37–53)	45 (37–53)	44 (38–51)	0.55
Screening history				0.90
Due (3–4 years)	193 (68.2)	140 (67.6)	53 (69.7)	
Overdue (≥5 years)	61 (21.6)	46 (22.2)	15 (19.7)	
Never screened	29 (10.2)	21 (10.1)	8 (10.5)	
Years since last screened (median, IQR)	n = 254	n = 186	n = 68	0.82
	3.9 (3.4–4.9)	3.9 (4.4–5.0)	4.0 (3.4–4.7)	
Time from when the kit was sent to when the sample was collected in days (median, IQR)	-	22 (10–48)	-	-

* *p*-value compares participants who did and did not return kits.

**Table 2:** HPV positivity by screening history.

	Total N = 202	Due (3–4 Years) N = 140	Overdue (≥5 Years) N = 42	Never Screened N = 20
HPV Positive	15	11	3	1
	(7.4%, CI: 4.5–12.0)	(7.8%, CI: 4.3–13.6)	(7.1%, CI: 1.8–19.7)	(5.0%, CI: 0.9–25.4)

Table 2 totals only include participants with valid HPV results.

**Table 3:** Demographics of participants who completed the post-participation survey.

	Returned Kit and Fully Completed Survey N = 42 *
Age at kit sent (median, IQR)	48 (40–54)
Ethnicity (n = 39)	
Asian	25 (64.1%)
Indigenous	1 (2.6%)
White	12 (30.8%)
Other/Multiethnic	1 (2.6%)
Languages can comfortably communicate in (*check all that apply*)	
English	35 (83.3%)
Punjabi	11 (26.2%)
Hindi	10 (23.8%)
French	1 (2.4%)
Mandarin	1 (2.4%)
Tagalog	1 (2.4%)
Other	2 (4.8%)
Prefer not to answer	1 (2.4%)
Household income in 2018 (n = 29)	
Under CAD 30,000	2 (6.9%)
CAD 30,000–59,999	6 (20.7%)
CAD 60,000–89,999	7 (24.1%)
Over CAD 90,000	14 (48.3%)

* Survey results in this table include only those who returned kits and completed the entire survey. There were 7 incomplete surveys and 2 participants who did not return kits completed the survey who have been removed from survey analysis.

**Table 4:** Participant experience using the website and self-screening.

**Reasons for participating in CervixCheck *(check all that apply)***	
Convenience	27/51 (52.9%)
Time	20/51 (39.2%)
Stress	16/51 (31.4%)
Comfort	22/51 (43.1%)
Other	5/51 (9.8%)
**Experience of using the CervixCheck website**	
Did the CervixCheck website provide enough information about cervical cancer and HPV to answer any questions?	Yes
	39/45 (86.7%)
Did the CervixCheck website provide enough information about participating in the project?	Yes
	42/46 (91.3%)
Signing up online for a CervixCheck account was…	Easy/Very Easy *
	43/46 (93.5%)
It was easy to use the website	Agree/Strongly Agree
	37/44 (84.1%)
The language used on the website was easy to understand	Agree/Strongly Agree
	38/43 (88.4%)
Felt confident about the confidentiality and privacy	Agree/Strongly Agree
	36/43 (83.7%)
Were satisfied with the content and features of the website	Agree/Strongly Agree
	35/43 (81.4%)
**Experience of HPV self-screening** ***Among participants who completed self-screening***	
The instructions in the kit were easy to follow	Agree/Strongly Agree
	35/38 (92.1%)
Self-collecting ^†^ a cervical sample was easy to perform	Agree/Strongly Agree
	33/38 (86.8%)
I felt that I was collecting the sample correctly	Agree/Strongly Agree
	33/38 (86.8%)
The self-collection device was comfortable to use	Agree/Strongly Agree
	35/38 (92.1%)
Did you experience any pain or discomfort that discouraged you from self-screening?	No
	35/38 (92.1%)
**Receiving results online** ***Among participants who completed self-screening***	
How did you feel about receiving your screening results online through the CervixCheck website?	Satisfied/Very Satisfied
	33/38 (86.8%)
Did you understand your online results?	Yes
	35/38 (92.1%)
**Future screening intentions and recommendations** ***Among participants who completed self-screening***	
Assuming that both HPV self-collection and having a healthcare provider collect a cervical sample are equally safe and effective for testing, what would you prefer in a future screening program?	
Self-screening	32/38 (84.2%)
Sample taken by doctor	2/38 (5.3%)
No preference	4/38 (10.5%)
If you had the opportunity, how likely are you to use self-collection again in the future for cervical cancer screening?	Likely/Very Likely
	35/38 (92.1%)
How likely are you to recommend self-collection to other women you know?	Likely/Very Likely
	35/38 (92.1%)
**Preferred methods for receiving self-collection kits in the future**	
By signing up online through a website like CervixCheck	12/51 (23.5%)
By visiting my family doctor and getting the kit during my visit	7/51 (13.7%)
Automatically receiving one in the mail from the screening program when I am due for screening	28/51 (54.9%)
Having the option to request a kit be sent from the screening program when I am due for screening	14/51 (27.5%)

* For the Likert scale results, frequencies were aggregated for (1) Very Easy and Easy, (2) Strongly Agree and Agree, (3) Very Satisfied and Satisfied, and (4) Very Likely and Likely. ^†^ The term “self-collection” was used in the survey to describe self-screening.

## Data Availability

The datasets presented in this article are not readily available because the data are part of provincial medical data repositories with restricted access as well as due to technical limitations and the sensitive nature of these data. Requests to access the datasets should be directed to the corresponding author, Laurie Smith.

## Supplementary Materials

The following supporting information can be downloaded at https://www.mdpi.com/article/10.3390/curroncol31090399/s1, Document S1: Complete post-participation feedback survey.
