# Supplementary material for: Self-Screening for Cervical Cancer Offered through a Digital Platform in a Region of British Columbia with Lower Screening Rates
Source: Curr Oncol. 2024 Sep 13;31(9):5399–411. doi: 10.3390/curroncol31090399 (PMC11431596; doi:10.3390/curroncol31090399)
Supplement: Supplementary file 1 [file curroncol-31-00399-s001.zip › Supplementary Material_CervixCheck Survey.pdf]

CERVIXCHECK
cervical cancer screening at home

1. **What were your reasons for choosing to participate in CervixCheck (select all that apply)?**

   Convenience: Requesting a self-collection kit online and collecting my own sample was very convenient

   Time: I was able to save time by requesting a kit online and collecting my own sample

   Stress: Requesting a kit online and collecting my own sample was less stressful

   Comfort: It is physically uncomfortable for me to receive a Pap test and CervixCheck allowed me to more comfortably collect my own sample

   Other

   Prefer not to answer

   You selected "Other". Please provide us with your reason(s) for participating in CervixCheck below:

   R: ____

2. **Prior to participating in CervixCheck, had you ever been screened for cervical cancer (e.g. Pap test)?**
   - Yes
   - No
   - Don't know

3. **Did you self-collect and return your self-collection device?**
   - Yes
   - No

4. **Signing up online for a CervixCheck account was:**
   - Very easy
   - Easy
   - Neutral
   - Difficult
   - Very Difficult
   - Not sure, someone helped me

5. **What type of device did you use to sign-up for an account?**
   - Desktop computer/laptop
   - iPad/Tablet
   - Mobile phone
   - Don't know

6. **Did the CervixCheck website provide you with enough information about the project?**
   - Yes

- No, I still had questions
- Don't know

7. **You answered "No" to the above question. Do you have any suggestions for what information could have been included?"**

   R: ___________________________________

8. **Did the CervixCheck website provide enough general information about cervical cancer and HPV to answer any questions?**
   - Yes
   - No, I still had questions
   - Don't know

9. **You answered "No" to the question above. What information could have been included?"**

   R: ___________________________________

10.

	**Strongly Disagree**	**Disagree**	**Agree**	**Strongly Agree**	**Don't Know**
I found it was easy to use the CervixCheck website					
The language used on the CervixCheck website was easy to understand					
I felt confident about the confidentiality and privacy measures built into the CervixCheck website					
I was satisfied with the content and features of the CervixCheck website					

11. **Assuming self-collection kits could be available in the future, how would you prefer to receive a kit?**
    - By signing up online through a website like CervixCheck
    - By visiting my family doctor and getting the kit during my visit
    - Automatically receiving one in the mail from the screening program when I am due for screening
    - Having the option to request a kit to be sent from the screening program when I am due for screening

- No preference
- Don't know

12. Do you have any additional comments or thoughts you would like to share about the CervixCheck website? Please state in the space provided.

R: ____

13.

	Strongly Disagree	Disagree	Agree	Strongly Agree	Don't Know
The instructions in the self-collection kit were easy to follow					
Self-collecting a sample was easy to perform					
Using the self-collection device, I felt confident that I was collecting the sample correctly					
The self-collection device was comfortable to use					

14. Did you experience any pain or discomfort that discouraged you from self-collecting?

- Yes
- No
- Don't know

15. Did you experience any other difficulties with self-collection?

- Yes
- No
- Don't know

16. Is there anything you would like to share with us about your experience with self-collection?

R: ______________________________________

17. If you had the option of participating in online cervical cancer screening again (for example, CervixCheck) instead of having a Pap test, how likely are you to participate in online screening?

- Very likely
- Somewhat likely

- Not likely
- Very unlikely
- Don't know

18. Assuming that both HPV self-collection, and having a health care provider collect a cervical sample are equally safe and effective for testing, what would you prefer in a future screening program?
- Self-collection
- Sample taken by a physician
- Either way, I have no preference
- Don't know
- Prefer not to answer

19. If you had the opportunity, how likely are you to use self-collection again in the future for cervical cancer screening?
- Very likely
- Somewhat likely
- Not likely
- Very unlikely
- Don't know

20. How likely are you to recommend self-collection to other women you know?
- Very likely
- Somewhat likely
- Not likely
- Very unlikely
- Don't know

21. Do you have any additional comments or thoughts about self-collection that you would like to share below?

R: ____

Questions 22-28 only apply to participants who answered "Yes" to Question 3.

22. How did you feel about receiving your screening results online through the CervixCheck website?
- Very satisfied
- Satisfied
- Neutral

- Dissatisfied
- Very dissatisfied
- Don't know

23. Did you understand your online results?

- Yes
- No
- Unsure

24. If your online results showed that you did not have HPV (i.e. normal results), was it clear to you when and where you should return for your next screening?

- Yes
- No
- Don't know
- Not applicable

25. If your online results instructed you to contact your doctor, how did you feel about the way your results and instructions for follow-up were delivered?

- Very satisfied
- Satisfied
- Neutral
- Dissatisfied
- Very dissatisfied
- Not applicable
- Prefer not to answer

26. Your doctor may have recommended follow-up appointments based on your HPV test results. Did you attend any recommended follow-up appointments (either Pap test or Colposcopy)?

- Yes
- No
- I am not sure if I needed follow-up
- Not applicable

27. If you answered "No" to question 26, please state your reason for not attending follow-up below.

R: ____

28. Do you have any additional comments about how you received your HPV screening results? Please state in the space provided.

R: ____

Demographics

29. How did you hear about CervixCheck? (check all that apply)

Family physician
Medical office assistant/receptionist
Clinic poster
CervixCheck Study team member
Another study participant
Family/friend
Other

30. Which best describes your current gender identity?

- Female
- Male
- Woman
- Man
- Indigenous or other cultural identity (e.g. Two-Spirit)
- Something else (e.g. gender fluid, non-binary)

31. What sex were you assigned at birth?

- Male
- Female
- Intersex
- Prefer not to answer

32. How old are you?

- 30 – 39 years of age
- 40 – 49 years of age
- 50 – 59 years of age
- 60 – 65 years of age
- 65+ years old
- Prefer not to answer

33. Do you identify as an Indigenous person from North America?

- No, I do not identify as an Indigenous person from North America
- Yes, First Nations
- Yes, Métis
- Yes, Inuk (Inuit)
- Prefer not to answer

34. You may belong to one or more racial or cultural groups on the following list. Please check all that apply:

- o South Asian (e.g. East Indian, Pakistani, Sri Lankan)
- o Southeast Asian (e.g. Vietnamese, Cambodian, Malaysian, Laotian)
- o White
- o Chinese
- o Filipino
- o Black
- o Latin American
- o Arab
- o West Asian (e.g. Iranian, Afghan)
- o Korean
- o Japanese
- o Other – Specify
- o Prefer not to answer
- o Don't know

35. What languages do you feel comfortable reading/speaking (select all that apply)?

Punjabi
Hindi
English
French
Mandarin
Cantonese
Tagalog (Filipino)
Vietnamese
Korean
Persian (Farsi)
Spanish
Other
Prefer not to answer

36. What was your 2018 household income after tax?

- o Under $5,000
- o $5,000 to $9,999
- o $10,000 to $14,999
- o $15,000 to $19,999
- o $20,000 to $24,999
- o $25,000 to $29,999
- o $30,000 to $34,999

- $35,000 to $39,999
- $40,000 to $44,999
- $45,000 to $49,999
- $50,000 to $59,999
- $60,000 to $69,999
- $70,000 to $79,999
- $80,000 to $89,999
- $90,000 to $99,999
- $100,000 to $149,999
- $150,000 and over
- Prefer not to answer

37. Is there anything that you would like to share to help improve CervixCheck in the future?

____
